# Benefit of Advanced 3D DSA and MRI/CT Fusion in Neurovascular Pathology

**DOI:** 10.1007/s00062-022-01260-0

**Published:** 2023-02-06

**Authors:** Tomas Dobrocky, Marco Matzinger, Eike I Piechowiak, Johannes Kaesmacher, Sara Pilgram-Pastor, Johannes Goldberg, David Bervini, Tomas Klail, Vitor Mendes Pereira, Werner Z’Graggen, Andreas Raabe, Pasquale Mordasini, Jan Gralla

**Affiliations:** 1 University Institute of Diagnostic and Interventional Neuroradiology, Inselspital, Bern University Hospital, University of Bern, Freiburgstr. 8, 3010 Bern, Switzerland; 2 Department of Neurosurgery, University of Bern, Inselspital, Bern University Hospital, University of Bern, Bern, Switzerland; 3 Divisions of Neurosurgery and Therapeutic Neuroradiology, St Michael’s Hospital, Toronto, ON Canada

**Keywords:** Image fusion, AVM, Rotational angiography, Aneurysm, Intracerebral hemorrhage

## Abstract

Digital subtraction angiography provides excellent spatial and temporal resolution; however, it lacks the capability to depict the nonvascular anatomy of the brain and spinal cord.

A review of the institutional database identified five patients in whom a new integrated fusion workflow of cross-sectional imaging and 3D rotational angiography (3DRA) provided important diagnostic information and assisted in treatment planning. These included two acutely ruptured brain arteriovenous malformations (AVM), a small superficial brainstem AVM after radiosurgery, a thalamic microaneurysm, and a spine AVM, and fusion was crucial for diagnosis and influenced further treatment.

Fusion of 3DRA and cross-sectional imaging may help to gain a deeper understanding of neurovascular diseases. This is advantageous for planning and providing treatment and, most importantly, may harbor the potential to minimize complication rates. Integrating image fusion in the work-up of cerebrovascular diseases is likely to have a major impact on the neurovascular field in the future.

**Video online:**

The online version of this article contains 3 videos. The article and the videos are online available (10.1007/s00062-022-01260-0). The videos can be found in the article back matter as “Electronic Supplementary Material”.

## Introduction

A thorough understanding of neurovascular anatomy is the key to differentiating normal from pathological findings, planning and providing appropriate treatment and, most importantly, for minimizing complication rates. Digital subtraction angiography (DSA) remains the gold standard for the study of many neurovascular disorders including aneurysms, arteriovenous malformations and dural arteriovenous fistulas. The technique combines an excellent spatial and temporal resolution and provides insight into the hemodynamics of the vascular territory being investigated; however, its drawback is the problem of visualizing complex three-dimensional structures, which mainly result from overlapping anatomy, and this may limit our understanding. This led to the introduction of 3D rotational angiography (3DRA) to allow better visualization of complex intracranial anatomy. This technique may also help to find a suitable working projection during interventional procedures; however, neither of these techniques has the capability to depict the vessel wall, and only a limited capacity to depict adjacent brain parenchyma or spinal cord. On the other hand, cross-sectional imaging, especially magnetic resonance imaging (MRI), provides a high contrast of the brain and spinal cord. Recently, new angiographic systems providing an integrated workflow for image fusion during neurointerventional procedures have been introduced.

The goal of this study was to present advanced fusion techniques in exemplary cases and discuss how their integration into routine clinical practice may enable a deeper understanding of neurovascular disease and provide crucial information for treatment planning.

## Methods

Recently, a new angiographic system (ARTIS icono; Siemens Healthineers, Erlangen, Germany) was introduced in our neuroradiology department in Bern. The system offers the possibility to fuse 3DRA and cross-sectional image datasets using the integrated software. A review of the institutional database was performed to identify all patients treated between August 2021 and January 2022 in whom fusion of cross-sectional imaging and 3DRA had provided important diagnostic information and assisted in the treatment planning.

All patients provided a written, dedicated consent according to the Swiss Federal Data Protection Act. The study was performed in accordance with the guidelines of the institutional review board.

To obtain the best possible results, isotropic images of any arbitrary computed tomography (CT) or MRI scan could be used. DSA was performed on a biplane high-resolution angiographic system (ARTIS icono). The system uses an as40HDR flat-panel detector with a 49 cm diagonal entrance plane and an active imaging size of 398 × 293 mm and an active matrix size of 2586 × 1904 pixels. Standard transfemoral access was used to catheterize all supra-aortic arteries supplying the brain with a 5 Fr catheter. A full four-vessel angiogram in standard anteroposterior and lateral projections was acquired first. For 3DRA the diagnostic catheter was placed at the origin of the artery supplying the intracranial vascular lesion of interest. A 4‑s 3DRA was then performed focusing on the lesion of interest using the same flat-panel detector system.

### Fusion Process

The fusion was performed using the dedicated fusion software (syngo Application Software, Siemens Healthineers, Erlangen, Germany) provided by the manufacturer. The process was performed by one interventional neuroradiologist (TD, 10 years of experience), and one experienced radiology technician (MM, 11 years of experience). The 3DRA of an injected vascular territory and the cross-sectional images were loaded into the dedicated workspace. To achieve the most accurate results the two volumes need to be aligned. This can be done either by automatic registration or manually by shifting and rotating one volume to match the positions of the anatomical structures of the other volume. Finally, verification of accurate alignment of both volumes was performed in the multiplanar mode focusing on important landmarks such as major arteries and veins, bony structures, or the brain and spinal cord parenchyma.

To improve the visualization and enhance the understanding of the neurovascular target lesion, segmentation and windowing were performed on a case by case basis. The threshold method was used to assign a specific color to vascular structures of interest. In non-shunting lesions all opacified vessels of the injected territory were displayed in red on 3DRA. For shunting lesions, two distinct colors were used: red for the artery demonstrating higher Hounsfield unit (HU) values and purple or blue for the vein demonstrating lower HU values. If embolization was performed, the liquid embolic agent was displayed in green.

To improve visualization in specific cases the embedded multiplanar reconstruction mode was used. This mode allows embedding of the reconstructed vessels derived from the 3DRA with the volume rendering technique on an arbitrary cross-sectional image.

Blending of datasets was performed individually to enhance the visualization of the target lesion. Snapshots and multiplanar reconstructions were exported to the local storage facility. Generally, maximum intensity projections were not reconstructed as this may decrease image quality.

### Illustrative Cases

#### Patient 1

**Fig. 1:**
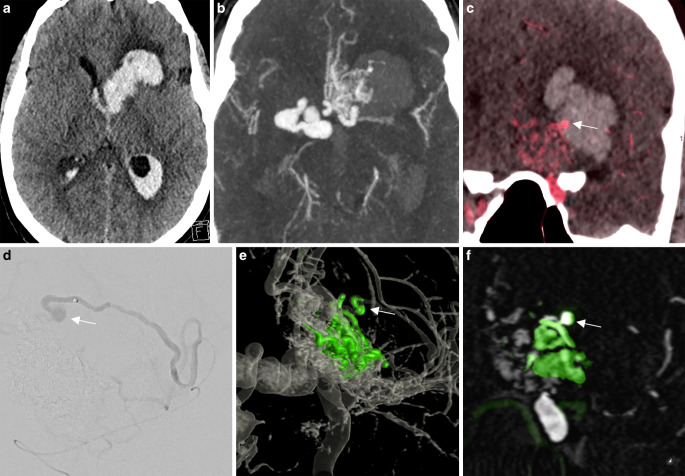
Ruptured arteriovenous malformation (AVM) with an intranidal aneurysm. **a** Nonenhanced computed tomography (CT) in the axial plane demonstrating a deep left basal ganglia hemorrhage with intraventricular rupture. **b** Computed tomography angiography (CTA) demonstrating a nidus in the left orbitofrontal cortex with several enlarged venous pouches on the contralateral side overlying the carotid terminus. **c** Three-dimensional rotational angiography (3DRA) + CT fusion in the coronal plane demonstrating a small intranidal aneurysm (*arrow*) centered in the basal ganglia hemorrhage as the bleeding source. **d** Selective catheterization of the deep perforating branch supplying the aneurysm (*arrow*). **e**,**f** Baseline 3DRA + postembolization 3DRA fusion demonstrating the onyx cast (*green*). Note that the inferior and lateral part of the AVM with the aneurysm (*arrow*) have been embolized. Supplementary video demonstrates the fusion of baseline 3DRA + postembolization 3DRA in axial slides

A middle-aged female presented with a left basal ganglia hemorrhage with intraventricular rupture on admission (Fig. 1). The CTA demonstrated a small nidus in the left orbitofrontal cortex with several enlarged venous pouches on the contralateral side overlying the carotid terminus. The DSA confirmed the diagnosis of an arteriovenous malformation (AVM), which was mainly supplied by a frontal branch originating from the left A2 segment and multiple enpassant feeders from the ipsilateral A1 and M1 segment. Fusion of the non-enhanced CT scan and a 3DRA clearly showed a small intranidal aneurysm located at the center of the basal ganglia hemorrhage as the source of the bleeding. In the acute setting, the decision for targeted embolization of the intranidal aneurysm was made.

Originally, a superselective embolization of the aneurysm through the frontal branch supplying the lateral part of the AVM was intended. Because of insufficient penetration of the aneurysm, a deep perforator originating from the left M2 segment was selectively catheterized and the aneurysm was occluded with ethylene vinyl copolymer (EVOH).

The fusion of the pre-embolization and post-embolization 3DRA demonstrated occlusion of the lateral and inferior part of the AVM as well as the intranidal aneurysm with EVOH (Fig. 1e, f, green). The fusion technique enables better visualization of the different compartments of the AVM; a frontobasal vein draining through the cribriform plate has been occluded with EVOH. The medial part of the AVM remains open and drains via a single posterior vein into multiple enlarged venous pouches on the right side (Video 1).

#### Patient 2

**Fig. 2:**
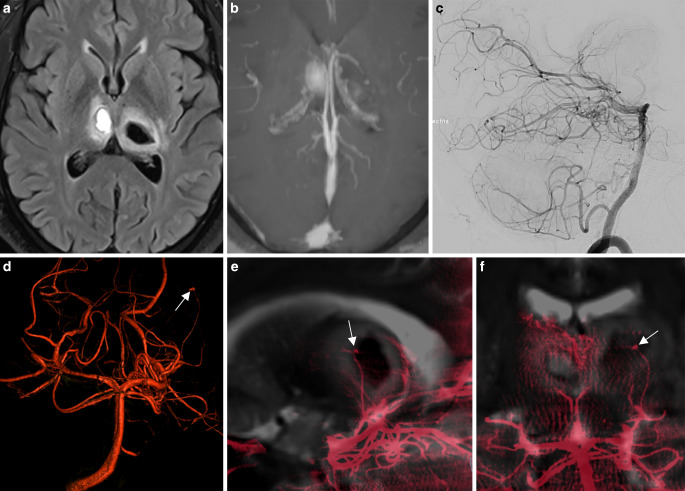
Ruptured thalamo-perforating branch micro-aneurysm. **a** Baseline magnetic resonance imaging (MRI) demonstrating a bithalamic hemorrhage. **b** No sign of central venous thrombosis. **c** Digital subtraction angiography (DSA) run of the posterior circulation in the lateral projection with normal findings. **d** Three-dimensional rotational angiography (3DRA) showing a small aneurysm (*white arrow*) in the course of a left thalamo-perforating branch. **e**,**f** 3DRA + MRI fusion clearly demonstrates the aneurysm located within the left thalamic hemorrhage as the bleeding source

A middle-aged male patient with multiple vascular risk factors was admitted with dizziness, and headache. The baseline MRI acquired on admission demonstrated bithalamic and central pontine hemorrhages and multiple small diffusion-restricted areas in the supratentorial white matter (Fig. 2). Dedicated sequences demonstrated vessel wall enhancement of multiple middle-sized and small intracranial arteries, and no sign of central venous thrombosis. Vasculitis screening including blood and cerebrospinal fluid samples failed to detect vasculitis biomarkers, and no signs of endocarditis were seen.

The DSA demonstrated caliber irregularities of multiple intracranial vessels and no arteriovenous shunt. A 3DRA demonstrated a small aneurysm (2 mm) in the course of a left thalamo-perforating branch with vague contact to the thalamic hemorrhage. Fusion of the T2-SPACE and a 3DRA clearly demonstrated the aneurysm location within the left thalamic hemorrhage. Because of multiple diffusion restrictions in different vascular territories, vessel wall enhancement, and vessel wall irregularities of medium and small caliber arteries, even in the absence of cerebrospinal fluid biomarkers, vasculitis was considered the most likely diagnosis. Due to the small vessel caliber (0.3 mm), superselective catheterization of the thalamo-perforating artery was unsuccessful. The patient made a good recovery after intensive cortisone treatment. Follow-up imaging demonstrated resorption of the thalamic hemorrhage.

#### Patient 3

**Fig. 3:**
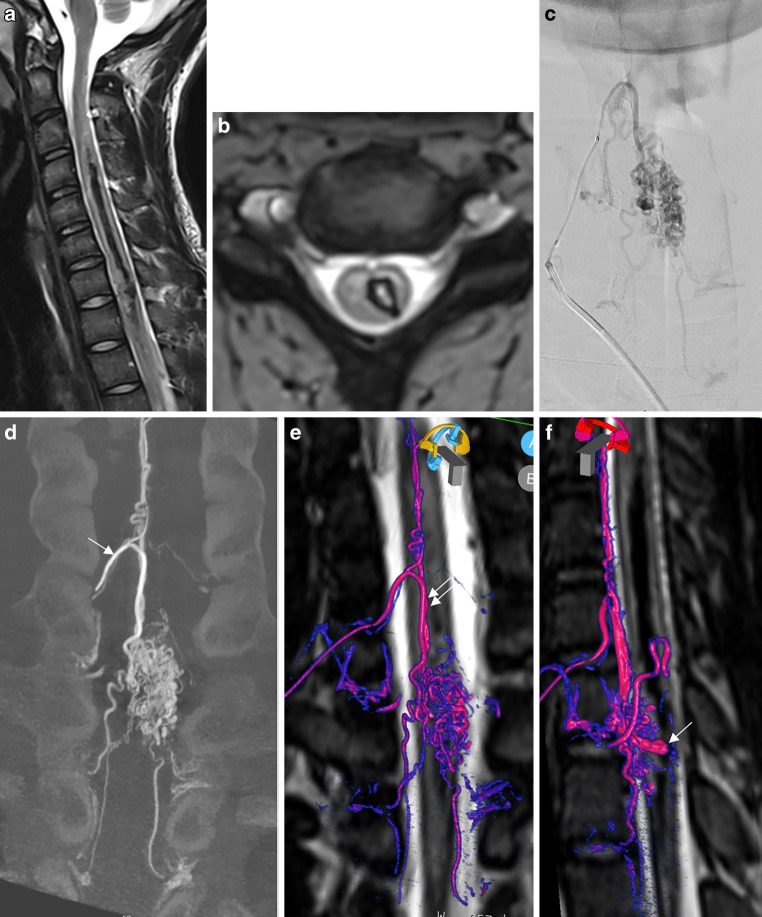
Ruptured spinal arteriovenous malformation (AVM) with a large intranidal aneurysm. **a** T2W sagittal image demonstrating extensive left-sided hematomyelia. **b** Hemoflash demonstrating the extension of the hematoma within the left side of the spinal cord. **c** (DSA anterio posterior projection). **d** (coronal reformated 3DRA) Superselective injection of the main radiculomedullary artery of the cervical enlargement (artery of Lazorthes, *arrow*) demonstrating a large nidus and multiple draining radicular veins. **e**, **f** Fusion of the three-dimensional rotational angiography (3DRA) and T2W spine magnetic resonance imaging (MRI) demonstrating the feeding radiculomedullary artery which supplies the anterior spinal artery (*double arrow*) which than feeds the AVM (*red/pink* for the artery and *purple* or *blue* for the draing vein). The intranidal aneurysm is localized within the central part of the myelon and the tip of the aneurysm is pointing towards the hematoma. Supplementary video demonstrates the fusion with an embedded 3D model of the AVM

A teenager with sudden left-sided hemiparesis was admitted. A spine MRI demonstrated left-sided hematomyelia and multiple flow voids localized within the spinal cord and on the pial surface at the cervicothoracic junction suggestive of a spinal AVM (Fig. 3). A DSA confirmed this diagnosis. The main contributor to the AVM was the radiculomedullary artery of the cervical enlargement (artery of Lazorthes) originating from the right deep cervical artery. Another radiculomedullary artery originating from the supreme intercostal artery was supplying the inferior part of the AVM. A 4 mm saccular aneurysm located on the medial border of the nidus was depicted on DSA. The fusion of a 3DRA and an isotropic T2w spine MRI demonstrated the aneurysm within the central part of the spinal cord, the tip of the aneurysm is located within the hematoma (Video 2). In the acute stage, targeted embolization of the aneurysm was performed with coils to prevent rebleeding. (DSA anterio posterior projection). The patient has made a promising clinical recovery and apart from a subtle weakness of the right arm remains asymptomatic.

#### Patient 4

**Fig. 4:**
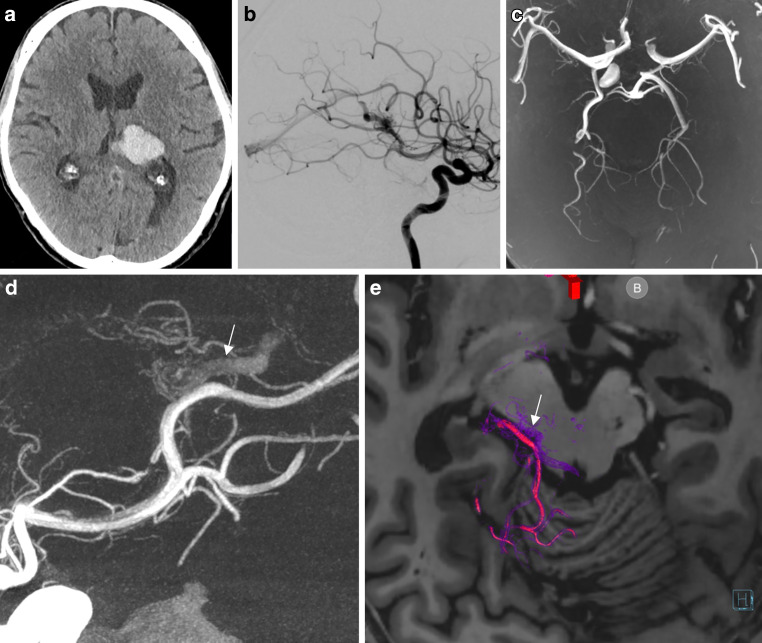
Small arteriovenous malformation (AVM) on the superficial surface of the mesencephalon after radiosurgery. **a** The baseline computed tomography (CT) scan demonstrating a left thalamic hemorrhage. **b** Digital subtraction angiography (DSA) showing the nidus and en-passant feeders from the left P3 segment and deep venous drainage. **c** Three years after radiosurgery, 7‑Tesla magnetic resonance imaging (MRI) does not show evidence of any abnormal vessels on the arterial time-of-flight sequence. **d** DSA demonstrates a residual arteriovenous shunt with early arterial filling of the posterior segment of the basal vein of Rosenthal (*arrow*). **e** Fusion of the follow-up 7‑Tesla and three-dimensional rotational angiography (3DRA) volumes clearly demonstrates the small nidus localized on the superficial surface of the posterolateral mesencephalon with a single draining vein corresponding to the posterior segment of the basal vein of Rosenthal (*purple*). Supplementary video demonstrates the fusion with an embedded 3D model of the AVM

A 68-year old male with right hemiparesis was admitted to our hospital. The baseline CT scan demonstrated a left thalamic hemorrhage with suspicious vessels in the ambient cistern (Fig. 4). The subsequent DSA demonstrated a left thalamic AVM fed by small en-passant feeders from the left P3 segment and deep venous drainage. The multidisciplinary board decided to refer the patient for radiosurgery and he was treated with 15 Gy. Follow-up 7‑Tesla MRI 3 years afterwards demonstrated a small parenchymal defect in the left posterior thalamus and no evidence of abnormal vessels on the arterial time-of-flight sequence; however, DSA demonstrated residual arteriovenous shunt with early arterial filling of the posterior segment of the basal vein of Rosenthal. The exact location of the nidus was not identifiable based on cross-sectional imaging.

Fusion of the follow-up 7‑Tesla and 3DRA demonstrates the location of the small nidus on the superficial surface of the posterolateral mesencephalon with a single draining vein corresponding to the posterior segment of the basal vein of Rosenthal (purple) (Video 3).

#### Patient 5

**Fig. 5:**
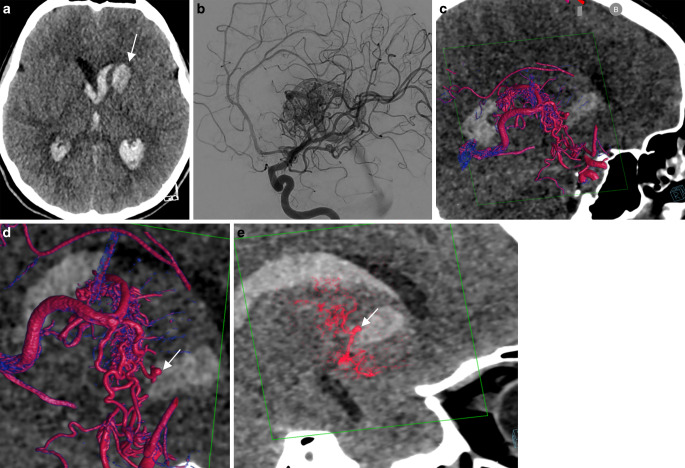
Ruptured periventricular arteriovenous malformation (AVM) with an intranidal aneurysm. **a** The non-enhanced computed tomography (CT) demonstrates a hemorrhage in the left caudate nucleus (*arrow*) with intraventricular rupture. **b** The digital subtraction angiography (DSA) shows a left basal ganglia nidus supplied by the anterior choroidal artery, draining via a single vein into the deep venous system. **c**,**d** Fusion of the non-enhanced baseline CT scan and three-dimensional rotational angiography (3DRA) shows a small intranidal aneurysm centered in the left caudate nucleus as the bleeding source. **e** Fusion of the baseline CT and superselective 3DRA with the microcatheter placed in the feeding artery supplying the aneurysm and the anterior part of the nidus

A teenage girl presented with sudden headache and decreased consciousness (Fig. 5). The non-enhanced CT demonstrated an intracerebral hemorrhage in the left caudate nucleus with intraventricular rupture. The DSA showed a left periventricular AVM supplied by the anterior and posterior choroidal artery branches and several hypertrophic lenticulostriate arteries. The nidus was draining into the deep venous system via a single outflow vein. Fusion of the non-enhanced baseline CT scan and the 3DRA showed a small intranidal aneurysm centered in the left caudate nucleus as the source of the bleeding. A microcatheter with a detachable tip was then navigated into a lenticulostriate artery branch supplying the aneurysm. A fusion of the baseline CT and superselective 3DRA via the microcatheter demonstrated the lenticulostriate artery supplying the rostral part of the nidus and the aneurysm, but no supplying branches to the basal ganglia. The aneurysm was successfully occluded with a liquid embolic agent.

## Discussion

The additional information gained from the fusion of 3DRA and cross-sectional imaging may be crucial in the diagnostic work-up of neurovascular pathologies, helping to guide decision-making and thus may have an important impact on patient management. This is particularly true when faced with complex neurovascular anatomy, where fusion can help to precisely visualize the spatial relationship between neurovascular structures and the brain parenchyma or spinal cord.

The 3DRA has been a gold standard in the neurointerventional field for several years [1]. It provides excellent spatial resolution of the vessel lumen in a specific vascular territory after injection of a contrast dye in the target vessel (luminography); however, due to the insufficient contrast to visualize structures surrounding the vessel lumen, like the vessel wall or brain parenchyma, our understanding may be limited. CT angiography and MR angiography are alternative, non-invasive techniques to visualize neurovascular anatomy. Although image quality of CTA and MRA has greatly improved, it remains inferior to DSA for visualizing medium and small sized vessels and does not provide information on the hemodynamics of a single vascular territory.

Image fusion is not entirely new to the neurovascular field, and various techniques have been reported in the literature. The first approach fuses two independently acquired 3DRAs to concurrently visualize two different arterial territories and thus provide a complete anatomical picture of a vascular anatomy [2]. A second approach fuses an arterial and a venous 3DRA of the same arterial territory, which may help to distinguish the venous drainage of an AVM or dural AVF from the venous drainage of the adjacent brain parenchyma [3]. Fusion of 3DRA and 4DRA (time-resolved 3DRA) and MRI of intracranial aneurysms and brain AVM has also been reported [4–6].

Unlike in a study that demonstrated perforating arteries originating near or from the aneurysm with the fusion and did not influence the treatment itself [7], the results provided by the fusion technique in the cases presented here had an important impact on further management. The usefulness of 3DRA and cross-sectional image fusion has also been reported by Mathusami et al. in pediatric cerebrovascular disease and for accurate flow-diverter placement [8, 9].

The fusion technique has improved substantially and the new generation angiography systems provide high-quality, readily available images unattainable by earlier systems. This technique pushes the limits of visualizing neurovascular anatomy, while similar images were previously only available with the help of medical illustrators. The benefit of these illustrations goes beyond a mere visualization to increase our understanding of neurovascular pathology and thus has an important impact on decision making.

The fusion workflow integrated on the angiosuite console is straightforward to use. The automatic registration of both imaging datasets works reliably when dealing with intracranial vessels; however, fusion of both datasets of the spine usually requires manual adjustment to match the correct vertebral levels and to adjust for different patient positioning. After a brief training module on use of the console and available tools, a single fusion is available in approximately 5 min and thus may easily be implemented as part of the clinical routine. When used in the angiosuite we believe that this technique has the potential to guide interventional procedures in the near future.

As demonstrated in our case series, the fusion technique may help to identify the bleeding source in patients with complex neurovascular pathologies, such as brain or spine AVMs or intracranial aneurysms. The fusion images help to assess the vicinity of neurovascular structures to important anatomical landmarks and functionally important areas of the brain and spinal cord. This could be important for assessing the type of functional deficit that is to be expected after injury to specific arteries during endovascular embolization or surgery. It may also provide valuable information for precise planning of radiosurgery (as for patient 4).

Brain AVMs are a challenging heterogeneous group of vascular pathologies and the approach to treatment of unruptured AVMs remains particularly controversial. High-flow arteriovenous shunts, overlapping, often tortuous and dysplastic arterial feeders and veins, contribute to the overall complexity. The fusion of cross-sectional imaging and 3DRA has the potential to overcome the current limitations, identify potential risk factors for bleeding, and help to understand various AVM compartments.

The diagnosis and management of aneurysms originating from perforating arteries (i.e., lenticulostriate arteries, non-trunk basilar perforators, thalamoperforating arteries) remain a challenge; however, due to the improved imaging quality and superior spatial relation between the intracranial vasculature and the brain parenchyma the fusion technique may lead to a higher detection rate of this pathology (patient 2). They are usually small and demonstrated a high-rate of favorable outcome in patients managed conservatively [10–12].

In conclusion, the additional information provided by fusion of 3DRA and cross-sectional imaging may help to refine diagnoses to guide therapeutic decision-making and contribute to risk reduction when treating neurovascular pathology.

## Supplementary Information

Patient 1
Supplementary video demonstrates the fusion of baseline three-dimensional rotational angiography (3DRA) + post-embolization 3DRA in axial slides.

Patient 3
Supplementary video demonstrates the fusion with an embedded 3D model of the arteriovenous malformation

Patient 4
Supplementary video demonstrates the fusion with an embedded 3D model of the arteriovenous malformation

## Abbreviations

3DRA: Three-dimensional rotational angiography
AVM: Arteriovenous malformation
CT: Computed tomography
CTA: Computed tomography angiography
DSA: Digital subtraction angiography
EVOH: Ethylene vinyl copolymer
HU: Hounsfield unit
MRI: Magnetic resonance imaging
